# Special Issue “Corrosion in Concrete: Inhibitors and Coatings”

**DOI:** 10.3390/ma14206211

**Published:** 2021-10-19

**Authors:** Luigi Coppola

**Affiliations:** 1 Department of Engineering and Applied Sciences, University of Bergamo, 24044 Dalmine, Italy; luigi.coppola@unibg.it; Tel.: +39-035-2052054; 2 UdR “Materials and Corrosion”, Consorzio INSTM, 50121 Florence, Italy; 3 Consorzio Interuniversitario per lo Sviluppo dei Sistemi a Grande Interfase—Center for Colloid and Surface Science (CSGI), 50019 Sesto Fiorentino, Italy

The climatic changes that are taking place in recent years have increased awareness of the importance of environmental protection and the urgent need for industrial strategies aimed at a sustainable development. The construction industry has always been perceived as one of the sectors with the greatest environmental impact and therefore the cement and concrete production has developed several strategies to limit the environmental impact that largely derives from the binder production [1,2,3]. These strategies are aimed not only at reducing the environmental impact (carbon dioxide emissions, energy consumption and natural raw materials depletion) but also at improving the performance and durability of building materials. 

Penetration of aggressive external agents such as carbon dioxide or chlorides is mainly responsible for the deterioration of reinforced concrete structures [4,5,6]. For new buildings exposed to natural environments, the durability of concrete elements can be guaranteed with proper mix design and construction details; when the environmental exposure is severe (i.e., industrial environments) or in the case of existing structures with durability deficiency, these problems could be solved by using electrochemical techniques, cathodic protection, corrosion inhibitors and surface treatments. 

This Special Issue “Corrosion in Concrete: Inhibitors and Coatings” was launched to create new food for thought on the issue of durability of existing concrete structures and to collect the latest findings on the protection of reinforced concrete from corrosion of reinforcement and deterioration of cement matrix. Papers collected in this Special Issue are perfectly consistent with the proposed topic and, embracing different facets, provide the reader with different points of view. A brief summary is given below.

The paper by Coffetti et al. [7] reports—in an exhaustive and discriminating way—the main coating types available on the market, outlining a handbook to help in choosing the most suitable protective treatment as a function of the environment, the concrete properties and the job-site characteristics. The performance of polymeric and cementitious coatings, hydrophobic impregnations and pore blocking treatments were also analyzed in compliance with current standards and the different optimal uses for each protective were outlined from the comparison of the experimental results.

Jiang et al. [8] and Merachtsaki et al. [9] proposed innovative coatings for the protection of concrete against seawater and bio-corrosion, respectively. The first paper focuses on 85 geopolymer pastes containing fly ash, ground granulated blast-furnace slag, metakaolin and Portland cement able to ensure excellent adhesion to concrete (up to 3.4 MPa) and ultra-high resistance in seawater. The latter deals with the development of a magnesium hydroxide coating able to prevent the microbiologically induced corrosion of concrete sewer pipes. The prolonged protection of the coating was confirmed by using short and long duration accelerated sulfuric acid spraying tests and measuring the coating consumption with scanning electron microscope analyses, X-ray diffractions and attenuated total reflectance measurements. 

Coppola et al. [10] analyzed the behavior of a silane-based water-repellent migrant corrosion inhibitor applied on different concretes, focusing on the influence of the concrete composition (water-to-cement ratio, cement type, cement factor) and curing age on the effectiveness of the corrosion inhibitor. Experimental results evidenced the effectiveness of silane-based treatment in improving the durability of concrete exposed to chlorides both in accelerated and natural diffusion tests by means of a water repellent effect. 

Several authors have delt with the corrosion of the reinforcement bars in presence of chlorides, treating the subject from different points of view. Jasniok et al. [11,12] investigated the corrosion behavior and the bond strength of zinc-coated low-carbon reinforcing steel in chloride contaminated concrete in comparison with black steel. Experimental results evidenced the favorable impact of zinc coating on steel by providing two-year protection against corrosion in environments with high chloride content. On the contrary, it was noticed that the presence of zinc coating had a deleterious impact on the parameters of anchorage. Zhao et al. [13] proposed to pretreat the steel fiber with zinc phosphate to improve the resistance of fiber reinforced concrete against chlorides. It was evidenced that the phosphating treatment of fibers allows to enhance the bond strength between steel and concrete and improves the corrosion resistance of fibers without affecting their surface morphology. Finally, the paper by Garcia-Contreras et al. [14] studied the effect of impressed current cathodic protection on reinforced concrete manufactured with fly ash as cement replacement. Results evidenced that the microstructure of cementitious matrix strongly influenced the corrosion behavior of steel in concrete. 
